# Enzymatic and mRNA Transcript Response of Ovine 6-Phosphogluconate Dehydrogenase (6PGD) in Respect to Different Milk Yield

**DOI:** 10.1155/2010/512056

**Published:** 2009-11-16

**Authors:** Stamatina Trivizaki, George P. Laliotis, Iosif Bizelis, Maria A. Charismiadou, Emmanuel Rogdakis

**Affiliations:** Department of Animal Science, Laboratory of Animal Breeding and Husbandry, Agricultural University of Athens, Iera Odos 75, 118 55 Athens, Greece

## Abstract

Ovine 6-phosphogluconate dehydrogenase (6PGD) is an enzyme of the pentose phosphate pathway, providing the necessary compounds of NADPH for the synthesis of fatty acids. Much of research has been conducted both on enzymatic level and on molecular level. However, to our knowledge, any correlation between enzymatic activity and 6PGD gene expression pattern related to different physiological stages has not been yet reported. With this report, we tried to highlight if any correlation between enzymatic activity and expression of ovine 6PGD gene exists, in respect to different milk yield. According to the determined enzymatic activities and adipocytes characteristics, ewes with low milk production possessed a greater (*P* ≤ .001) 6PGD activity and larger adipocytes than the highly productive ewes. Although 6PGD expression pattern was higher in low milk yield ewes than in ewes with high milk production, this difference was not found statistically significant. Thus, 6PGD gene expression pattern was not followed by so rapid and great/sizeable changes as it was observed for its respective enzymatic activity, suggesting that other mechanisms such as post translation regulation may be involved in the regulation of the respective gene.

## 1. Introduction

6-Phosphogluconate dehydrogenase (6PGD) is an oxidative carboxylase that catalyzes the decarboxylating reduction of 6-phosphogluconate into ribulose 5-phosphate in the presence of NADP. This reaction is a component of the hexose mono-phosphate shunt and pentose phosphate pathways. 

The functional importance of the enzyme is generally recognized in providing NADPH for fat synthesis and ribose for nucleic acid synthesis [1]. In farm animals, fat synthesis affects the economic return of the producer [2]. Excess fat deposits influence negatively meat quality, grading of carcasses, and in high milk yield animals, their health status and future performance. 

Prokaryotic and eukaryotic 6PGDs are proteins of about 470 amino acids whose sequences are highly conserved. The amino sequences of almost 40 different 6PGDs have been reported including human [3], mouse [4], rat [5], and pig [6]. The protein is a homodimer in which the monomers act independently. Each contains a large, mainly alpha-helical, domain and a smaller beta-alpha-beta domain, containing a mixed parallel and antiparallel 6-stranded beta sheet. NADP is bound in a cleft in the small domain, the substrate binding in an adjacent pocket [7, 8]. 

In ruminants, extensive studies have been reported only for sheep and its cDNA. Carnet and Walker [9] were the first who determined the protein sequence of 6PGD reporting the isolation and characterization of 466 amino acids. However, this sequence information was incorrect due to misalignment of the peptides resulting during the protein determination. Somers et al. [10] revised the amino acid sequence based upon the isolation of the cDNA clones encoding the 6PGD gene in sheep. Thus, the isolated cDNA encodes a protein of 482 aa with a molecular mass of 52 kDa. The conservation of the protein sequence is very high as it shares an over 50% similarity with the protein encoded by the *E. coli* 6PGD gene and over 80% similarity with that of mammals (human, rodents, pig). 

It is known that in dairy ruminants major changes occur during lactation in the metabolism of several tissues such as adipose tissue, as part of the homeorhetic control of the organism. In many cases, as the animals are unable to consume sufficient energy during lactation, utilizes body reserves. During the last decades a lot of research has been conducted on enzymatic level showing the reaction of 6PGD activity in respect to different stimuli [2, 11–16] without, however, involving any study on molecular level (i.e., mRNA transcripts). Herein, we report for the first time the effect of different ovine milk yield of Chios breed ewes on the characteristics of adipocytes, on enzymatic activity and expression of 6PGD gene.

## 2. Materials and Methods

### 2.1. Animal Treatment

The experiment was carried out in the Experimental Station of the Agricultural University of Athens. Twenty Chios breed ewes aged 2–4 years old and with an average (±S.E) live weight of 55.4 ± 1.8 kg were randomly selected and divided in two groups according to their milk production after the weaning (40 ± 3 days post partum). Group A and Group B included animals with high (each animal possessed >1700 kg/day, *n* = 6) and low milk production (each animal possessed <1100 kg/day, *n* = 14), respectively. 

The ewes were milked by machine and were fed ad libitum twice daily, at 7:00 and 16:00 hour on an alfalfa hay and concentrated feed in pellets, designed to meet their maintenance and lactation requirements. Water was freely available. Milk samples were collected and fat content was determined according to Gerber method. Milk energy content was estimated in Mj of Net Energy according to the following equation [17]: *E* = [91.17 M(4.97 + *f*)]0.00418, where: *E* = milk energy (Mj), M = milk yield (kg/day), *f* = milk fat content  (%)*.*

Once per week samples of subcutaneous adipose tissue from tail region were taken, by biopsy. A day before sampling ewes fasted (24 hours) with free access in water, and in the day of sampling ewes were anaesthetized with the use of Zoletin 50 in 15–20 mg/kg BW. One sample (4–5 g) from adipose tissue was immediately frozen at −20°C for the determination of enzyme activity and lipid extraction. The second sample (0.5–1 g) was placed in Krebs-Ringer bicarbonate buffer (pH = 7.4, 37°C) for the measurement of adipocytes size and number, where as the third sample (~2.5–3.0 g) was immediately frozen in liquid N_2_ and stored at −80°C for further RNA extraction.

### 2.2. Biochemical Parameters

The diameter of 200 fat cells from each sample was measured as described by Rodbell [18]. The mean fat cell volume (*V*) of the 200 cells and mean adipocytes number/g adipose tissue were calculated from the mean diameter (*d*) and the standard deviation (*s*). The chemical fat content of adipose tissues was determined as described by Folch et al. [19]. 

For enzyme assay the method described by Rogdakis [20] was used. Enzyme activity was expressed as units per fat cell.

### 2.3. RNA Isolation and Reverse Transcription

Samples from tail subcutaneous adipose tissue were taken by biopsy. Total RNA from ovine adipose tissue was extracted using “Rneasy Lipid Tissue Kit” (Qiagen Cat no. 74804). For first-strand cDNA synthesis a two-step RT-PCR procedure was followed using 1 ug of the eluted total RNA, which was pre-treated with DNase I and Omniscript Reverse transcriptase (Qiagen) according to manufacturer's recommendations.

### 2.4. Semiquantitative RT-PCR Analysis

Total RNA from sheep adipose tissue was isolated as described above and used as template for a semi-quantitative RT-PCR analysis. For analysis, Ambion's QuantumRNA 18S Internal Standards Kit was employed, resulting in a 324 bp product. For 6PGD gene amplification the specific primers FP7: 5′-GGCCTACCACCTGATGAAGGACG-3′ and RP8: 5′-GCCAAATTCAGTTGCTGCCTGTC-3′ were used resulting in a 378 bp product. Multiplex PCR conditions were: 94°C for 3 minutes followed by 30 cycles each of 94°C for 30 seconds, 60°C for 30 seconds, 72°C for 30 seconds, with a final extension at 72°C for 2 minutes (Taq polymerase, New England Biolabs). The relative transcript amount was determined using the Scion Image software v. 4.0.3.2 (http://www.scioncorp.com/). Each sample was measured 4 times.

### 2.5. Statistical Analysis

Least Squares Procedures were employed in statistical analysis [21]. Fixed effects models were used to describe each individual observation concerning number and size of fat cells, enzymatic activity and transcriptional level of 6PGD that were affected by milk yield.

## 3. Results

Table 1 shows the average daily net milk energy (Mj), fat content (%), and milk production (kg). Group A possessed higher milk production (2.083 ± 0.108 kg) and higher net milk energy (8.545 ± 0.454 Mj, *P* ≤ .001) than Group B (0.715 ± 0.073 kg and 3.012 ± 0.289 Mj, respectively). Concerning the size of the observed adipocytes, the average diameter of adipocytes of all ewes was 74.03 ± 3.14 *μ*m. However, ewes of group A possessed statistically (*P* ≤ .01) smaller adipocytes than group B (64.38 ± 4.49 *μ*m versus 83.67 ± 2.94 *μ*m, resp.). 

An almost two fold greater number (*P* ≤ .01) of adipocytes per g of adipose tissue, was observed in ewes of group A in contrast to that of group B. The average number of adipocytes of tail adipose tissue of group A ewes was 4.58 ± 0.51∗10^6^/g adipose tissue while group B possessed 2.20 ± 0.34  ∗10^6^ adipocytes/g adipose tissue. 

**Figure 1:**
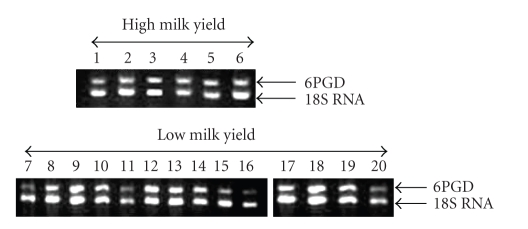
Expression analysis of 6PGD gene in tail adipose tissue of high and low milk yield ewes. All samples were normalized to 18S RNA gene. 10 *μ*L of each sample were loaded in the agarose gel for electrophoresis. As the experimental conditions were the same for all the samples, the observed differences in the low milk yield group may be due to differences concerns the sample itself.

**Figure 2:**
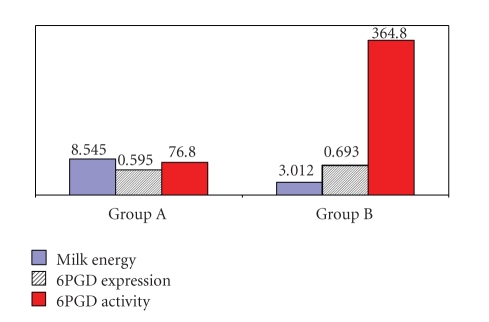
Correlation among enzymatic activity (nmol NADPH/10^6^ adipocytes), gene expression of 6PGD and milk energy (Mj) of lactating ewes.

The determined average enzymatic activity was significantly (*P* ≤ .05) greater in ewes of group B than that observed in group A (364.80 ± 49.73 nmol NADPH ∗ min^−1^/10^6^ adipocytes and 76.80 ± 13.50 nmol NADPH ∗ min^−1^/10^6^ adipocytes, resp.). Concerning 6PGD gene expression in the two groups during lactation **(** Figure 1) an increased transcript expression was observed in ewes of group B compared with the ewes of group A (0.693 ± 0.060 versus 0.595 ± 0.085, resp.). However, this difference was not statistically significant. Moreover, 6PGD expression did not follow the acute changes of enzymatic activity and milk energy level concerning the two groups (Figure 2).

## 4. Discussion

During lactation, udder is the tissue with the highest metabolic activity. In this study, the metabolic adaptations of tail adipose tissue of Chios ewes during milk production were of particular interest. 

Factors such as genotype, feed availability, body condition, number and weight of lambs at birth, environmental stimuli and their interaction may influence the daily milk production. According to Chilliard et al. [22], the mobilization of body fat depends on the initial body weight, as has been observed in sheep and dairy cows. As no differentiation of the body weight was observed during the study, the comparability of parameters between the examined groups was ensured. 

Regarding the number of adipocytes observed in this study, ewes of group A had approximately a two-fold increase of the observed number of fat cells/g adipose tissue in respect to that of group B ewes. The observed range of values regarding the number of fat cells observed in this experiment was significantly higher than the values recorded by previous researchers [11, 23, 24]. The difference is probably due to different productivity breeds of ewes (meat type breeds) and to the fact that the lambs in the experiments of Vernon et al. [23] and Travers et al. [24] have not been weaned. 

Comparing the size of fat cells in tail adipose tissue of the ewes it was observed that the fat cells were on average significantly smaller in ewes with high milk production than in ewes with low milk production. This result was expected since the highly productive ewes have greater energy requirements than that of ewes with low milk production and, thus, the pathway of lipolysis was more intensive in tail adipose tissue in order to cover the energy requirements of udder [25]. It is obvious that the catabolic activity of tail adipose tissue of ewes of group A in this study was higher as the quantity and the milk energy were increased. This observation is consistent with observations of Vernon et al. [23]. 

Diversification of lipogenic dehydrogenases activity such as 6PGD between animals with different milk productivity indicates the dependence of lipogenesis of ewes during lactation on the total energy content of the milk. In our study, the activity of 6PGD is reduced as the energy of the produced milk is increased (Figure 2). This observation was expected, as the ewes in group B due to the low milk production had lower energy requirements in order to meet the energy needs of the udder. In group A, a significantly less 6PGD activity was observed (Figure 2). In this case, the metabolic pathway of liposynthesis is limited, reducing thus the activity of lipogenic enzymes in order energy to be utilized for the maintenance of homeostasis of the body.

As shown in Figure 2, 6PGD activity in tail adipose tissue is reduced with the increase of milk energy. However, the difference in the values of 6PGD gene expression between the two groups was not statistically significant, although a decrease of expression was observed with the increase of milk production. The fact that the number of 6PGD gene transcripts between the two groups did not change significantly indicates that the control of 6PGD in adipose tissue during lactation may take place in translation and/or post-translation level, as previous authors have noted for important lipogenic genes during lactation, such as ACC [24, 26]. Moreover, in the low yield ewes group there seems to be an increasing rate of transcription of the 6PGD gene. This, may be due to more possible involvement of other mechanisms than the regulation of translation for example, the presence of a SNP, which might facilitate the rate of transcription (group B) or affect the post transcriptional events (group A). 

To sum up, the level of milk production significantly affects the metabolic role of tail subcutaneous adipose tissue. In ewes with high milk production, the pathway of lipogenesis is limited by reducing the size of fat cells and reducing the activity of 6PGD. In ewes with low milk production this restriction is less intense, as these animals have lower energy requirements compared to ewes with high milk production. The changes in the number of 6PGD gene transcripts in tail adipose tissue are not analogous either to the changes of 6PGD activity or to the level of milk production. 6PGD expression in the tail adipose tissue during dairy production is likely to be controlled through post-transcription and/or post-translation mechanisms. However, further research should be conducted in order to detect any potential relationship between gene expression, 6PGD activity and milk yield in sheep.

## Figures and Tables

**Table 1:** Average milk yield (kg), milk fat content (%) and net milk energy (Mj) of experimental ewes.

Group	Milk production	Fat content	Milk Energy
	(kg)	(%)	(Mj)
A (*n* = 6)	2.083a*** ± 0.108	5.867 ± 0.431	8.545*** ± 0.454
B (*n* = 14)	0.715^b^ ± 0.073	6.054 ± 0.176	3.012^b^ ± 0.289

Means with different superscript letters differ significantly (***P ≤ .001.)

## Abbreviations

aa: Amino acids
bp: Base pair
ACC: Acetyl coenzyme A carboxylase
BW: Body Weight
cDNA: DNA complementary to RNA
dNTPs: Deoxyribonucleotides triphosphate
6PGD: 6-phosphogluconate dehydrogenase
kDa: Kilodalton
MDH: Malate dehydrogenase
NADP: Nicotinamide adenine dinucleotide phosphate
NADPH: Reduced nicotinamide adenine dinucleotide phosphate
PCR: Polymerase chain reaction
RT: Reverse transcription.
